# Antimicrobial, mechanical and biocompatibility analysis of chlorhexidine digluconate-modified cements

**DOI:** 10.4317/jced.56308

**Published:** 2020-02-01

**Authors:** Gêisa-Aiane-de Morais Sampaio, Izaura-Helena-Chaves de Meneses, Fabiola-Galbiatti de Carvalho, Hugo-Lemes Carlo, Eliseu-Aldrighi Münchow, Taís-de Souza Barbosa, Matheus-Melo Pithon, Polliana-Muniz Alves, Rogério Lacerda-Santos

**Affiliations:** 1 DDS. M.Sc. Department of Clinical and Social Dentistry, Dental School, Federal University of Paraíba, João Pessoa, Paraíba, Brazil; 2 DDS., M.Sc., Ph.D. Professor. Department of Orthodontics and Pediatric Dentistry, Dental School, Federal University of de Fora, Governador Valadares, Minas Gerais, Brazil; 3 DDS., M.Sc., Ph.D. Professor. Department of Restorative Dentistry, Dental School, Federal University of de Fora, Governador Valadares, Minas Gerais, Brazil; 4 DDS., M.Sc., Ph.D. Professor. Department of Orthodontics, Dental School, State University of the Southwest of Bahia, Jéquie, Bahia, Brazil; 5 DDS., M.Sc., Ph.D. Professor. Department of Patology, Dental School, State University of Paraíba, Campina Grande, Paraíba, Brazil

## Abstract

**Background:**

The focus of this study was to evaluate the antimicrobial, mechanical properties and biocompatibility of glass ionomer (GICs) modified by Chlorhexidine (CHX).

**Material and Methods:**

For biocompatibility, 105 male Wistar rats were used, divided into 7 groups (n=15): Group C (Control,Polyethylene), Groups M, M10, M18, and Groups RL, RL10, RL18 (M-Meron and RL-Riva Luting: conventional, and modified with 10%, and 18% CHX, respectively). The tissues were analyzed under optical microscope for different cellular events and time intervals. Antibacterial effect and Shear Bond Strength Test (SBST) were also analyzed. Biocompatibility was analyzed by the Kruskal-Wallis and Dunn tests; SBST one-way ANOVA and Tukey test (*P*<0.05). For the antibacterial effect, the Kruskal-Wallis and Friedman, followed by Dunn (*P*<0.05) tests were used.

**Results:**

Morphological study of the tissues showed inflammatory infiltrate with significant differences between Groups C and RL18, in the time intervals of 7(*P*=0.013) and 15(*P*=0.032) days. The antimicrobial effects of the cements was shown to be CHX concentration-dependent (*P*=0.001). The SBST showed no significant difference between the Groups of Meron cement (P=0.385), however, there was difference between Group RL and Groups RL10 and RL18 (*P*=0.001).

**Conclusions:**

The addition of CHX did not negatively influence the SBST. Meron-CHX-10% was the most biocompatible, and Riva-CHX-18% had more influence on the inflammatory process and presented slower tissue repair.

** Key words:**Glass ionomer, chlorhexidine, biocompatibility, antimicrobial properties, microscope.

## Introduction

Orthodontic bands provide favorable conditions for the colonization of microorganisms (1). Glass ionomer cement(GIC) has important clinical properties such as fluoride release and adhesion to the dental structure (2), however, it does not have great antibacterial potential (1).

Antibacterial agents have been investigated so that when they are associated with the composition of GICs, they would be able to prevent demineralization of the enamel adjacent to orthodontic bands, result of bacterial colonization (3) and biofilm growth (4). Chlorhexidine digluconate (CHX) has been demonstrated to be efficient against bacterial species found in the oral cavity (5).

Authors have demonstrated that CHX in high concentrations could be toxic to the tissues (5). Added to this, GICs (6) may have cytotoxic effects resulting from its metallic components (7) that cause damage to gingival tissues (8).

Various attempts have been made to develop dental materials with antibacterial effect by means of adding CHX (9). However, the incorporation of CHX frequently resulted in changes in the mechanical and biological properties of materials, which could affects their clinical performance and tissue compatibility (1,9). Thus, the focus of this study was to analyze the antimicrobial, mechanical properties and biocompatibility of GICs with the addition of CHX.

## Material and Methods

-Experimental groups and animal model

Two GICs were used for cementation and contained 10% tartaric acid, Meron(Voco, Cuxhaven, Germany, Lot:1123187) and Riva Luting Plus (SDI, Victoria, Australia, Lot:10880571). Into another 2 solutions, which contained 10% or 18% chlorhexidine digluconate, the CHX was incorporated into them in drops in the proportion of 1:1 drops of tartaric acid/CHX by using the same dosing dropper, and the solutions were afterwards spatulated with the cement powder to obtain a solid material (10,11).

The animal experiment was approved by the Ethics Committee on Animal Research, Protocol/No.0582017. The sample size calculation was based on pilot study. For a standard deviation of 2.23 and a minimal intergroup difference of 5 for the inflammatory infiltrate to be detected, a sample of 5 animals was required to provide statistical power of 80% with an alpha of 0.05.

**Figure 1:**
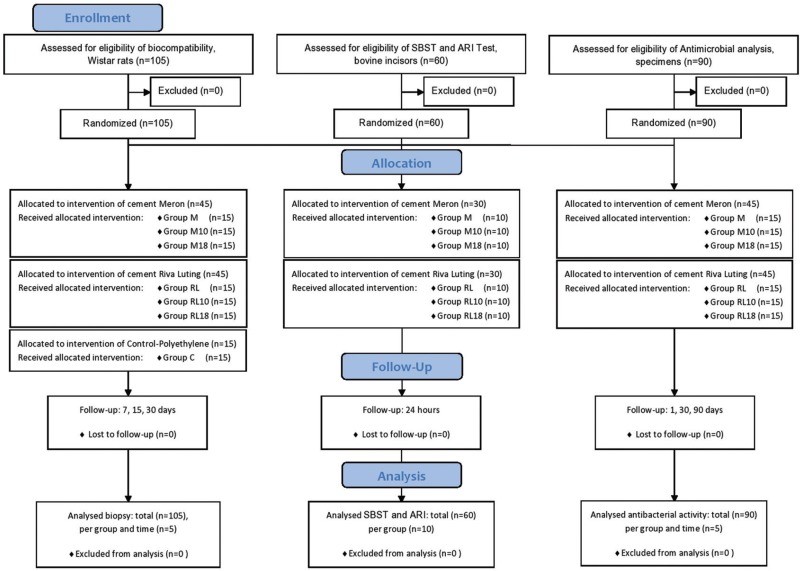
Flow Diagram of animals used, groups and tests evaluated.

In this study, 105 adult male Wistar rats with a mean weight of 250g were used. A total of 7 groups(n=15 rats for group) were created, as follows: Groups M, M10, M18 (Meron: conventional, and modified with 10% and 18% CHX respectively), Groups RL, RL10, RL18 (Riva Luting: conventional, and modified with 10% and 18% CHX respectively), and Group C (Control, Polyethylene) (1,11) were tested (Fig. 1).

Initially, the rats were anesthetized with an intraperitoneal injection of sodium thiopental (50mg/kg) (Cristália, SP, Brazil). Trichotomy was performed in the dorsal region of each animal, and for antisepsis, 4% chlorhexidine digluconate was used (12,13). On the midline, equidistant from the insertion of the animal’s tale and head, two incisions approximately 8 mm long by 18 mm deep were made. Each rat received two tube implants (1.5mm inner diameter X 5mm long) made of polyethylene (nontoxic Scalp Vein 19G). Previously, the tubes were autoclaved at a temperature of 120°C for 20 minutes and then used as inoculation vehicles for the tested materials (2).

The experimental materials were handled according to the manufacturers’ instructions. The GICs were introduced into the openings at the extremities of the tubes, using a syringe (Centrix, Connecticut, USA) supported on a glass slide at one extremity and a small glass slide at the other to flatten the material. For control Group, empty polyethylene tubes were used (2).

After the GICs had set, the tubes were implanted. The animals received 0.2 ml intramuscular dose of veterinary pentabiotic (Wyeth, New York, USA), and an injection of sodium dipyrone (0.3ml/100g, Novalgina, SP, Brazil). The animals were kept in individual cages under adequate conditions with appropriate rations and water ad libitum. After time intervals of 7, 15 and 30 days, the animals were anesthetized to obtain excisional biopsies, afterwards the rats were sacrificed by using a co2 chamber.

-Biocompatibility

The specimens, fixed in 10% formal, were prepared on glass slides by means of routine Hematoxylin and Eosin(HE) staining, and afterwards evaluated under an optical microscope Leica DM500® (Leica-Microsystems, Wetzlar, Germany), at 100x to 400x magnifications. The following histopathological parameters were evaluated: inflammatory infiltrate, edema, necrosis, granulation tissue, multinuclear giant cells, young fibroblasts and collagen fibers, and were awarded points according to the following scores:1–absent (when absent in the tissue); 2–scarce (when scarcely present, or in very small groups), 3–moderate (when densely present, or in some groups) and 4–intense (when found in the entire field, or present in large numbers). For each sample of the study, five sections representative of the histological condition of the tissue adjacent to the implanted materials were analyzed (1,11,14).

The histopathological evaluation was made by a single calibrated evaluator (Kappa=0.85). This was a randomized, triple-blind study; each material was directed to the groups I to VII, so that the examiner and statistician had no knowledge about the materials.

-Shear bond strength test-SBST and Adhesive remnant index-ARI

For the SBST test, 60(n=10) bovine incisors were used (Fig. 1). These were stored in a 0.1% Thymol solution until the time they were used for the experiment. The teeth were embedded vertical in PVC tubes (25x20mm) with acrylic resin(VIPI, SP, Brazil) (15). The vestibular surfaces of the crowns were positioned perpendicular to the base of the die at an angle of 90º. The vestibular surfaces were polished with a rubber cup (KG, Barueri, Brazil) and pumice stone(S.S.White,MG,Brazil) at low speed for 10 seconds, washed and dried for the same length of time (15).

Metal matrices(Morelli, Sorocaba, Brazil), measuring 4x5mm, were cut and metal brackets (Morelli, Sorocaba, Brazil) were welded onto them. The GICs were manipulated and each matrix was cemented in the center of the vestibular surface. After 5 min of initial setting time, the samples were stored at 37°C in relative humidity for 24h (10).

The SBST tests were performed in a universal test machine with a load cell of 10 Kg (EMIC-DL-200, Paraná, Brazil) using a chisel-shaped tip at a speed of 1mm/min. The SBST results were obtained in Kgf, transformed into N and divided by the bracket base area to provide results in MPa. After the test, the vestibular surface of each specimen was evaluated under a stereoscopic loupe (CarlZeiss, Göttingen, Germany) at 8x magnification, with the purpose of quantifying the ARI scores (16).

-Antimicrobial analysis

The antibacterial activity of the GICs was evaluated by the agar diffusion test, for which 90 specimens were used (n=15) (Fig. 1). The materials were inserted into polyethylene molds (6x3mm), left at 25°C for 5 min with the mold surfaces covered with a glass plate, and then stored at 37°C in 100% humidity for 60min. The samples were individually stored in 2mL of deionized water and stored for time intervals of 24h, 30 days and 90 days, with daily changes of water.

The bacterial strains of *Streptococcus mutans* ATCC-25175 culture stock were cultivated in brain heart infusion (BHI) (DIFCO-Becton, NJ, USA). The dilution of 10-1, containing 1.2x10-8CFU/ml was used, which was determined by means of serial dilution in 0.85% saline solution. After incubation at 37°C for 48h, the bacterial strain was spread on BHI agar plates and remained there at ambient temperature for 30 min. Subsequently, 3 samples (control, 10% and 18% of CHX) of the same GIC were placed on each agar plate in full contact between the samples and medium. After this, they were incubated at 37°C for 48h under microaerophilic conditions, and the diameters of the inhibition zones were measured with a digital pachymeter (Mitutoyo, Tokyo, Japan) in two planes-horizontal and vertical, in the time intervals of 24h, 30 days and 90 days.

-Statistical analysis

Distribution of the data was analyzed by the Kolmogorov-Smirnov test (GraphPad-Prism 5.0, San Diego, CA, USA). The results of the cellular events did not present normal distribution, therefore, they were submitted to the Kruskal-Wallis and Dunn test (*P*<.05). For SBST and ARI, ANOVA one-way and Tukey (*P*<0.05) tests were used. For the antibacterial effect, the Kruskal-Wallis and Friedman, followed by Dunn tests were used (*P*<0.05).

## Results

-Morphological study

**Figure 2:**
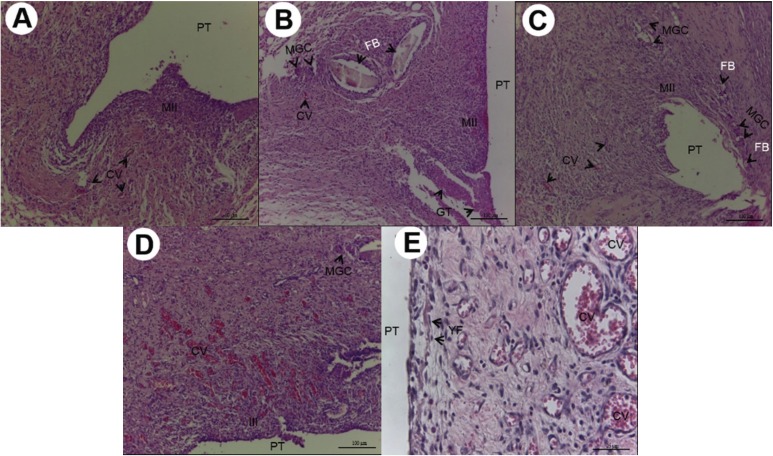
A) 7 days after implantation, Group M10:numerous and diminutive congested blood vessels (CV) associated with moderate inflammatory infiltrate (MII) (HE, 200X magnification, scale:50µm). Area of polyethylene tube implant (PT). B) 7 days after implantation, Group M18:moderate inflammatory infiltrate (MII), presence of multinucleated giant cells (MGC) close to the foreign body (FB), granulation tissue (GT) and congested blood vessels (CV) (HE,100X magnification, scale:100µm). Area of polyethylene tube implant (PT). C) 7 days after implantation, Group RL10: Cavity surrounded by moderate inflammatory infiltrate, congested blood vessels (CV),reaction of multinucleated giant cells (MGC) and foreign body (FB) (HE, 100X magnification, scale:100µm). Area of polyethylene tube implant (PT). D) 7 days after implantation, Group RL18: intense inflammatory infiltrate (III), congested blood vessels (CV), multinucleated giant cells (MGC) (HE, 100X magnification, scale: 100µm). Area of polyethylene tube implant (PT). E) 7 days after implantation, Group Control: vascularization with congested blood vessels (CV) of various sizes, in the midst of proliferation of young fibroblasts (YF) (HE, 400X magnification, scale: 25µm). Area of polyethylene tube implant (PT).

As regards the results of the morphological study, in the initial period, an intense inflammatory infiltrate was shown, particularly in Groups M18 and RL18, with statistically significant difference between the Control Group and Groups RL18 in the time intervals of 7 (*P*=0.013) and 15 (*P*=0.032) days ( Table 1). The intensity of the inflammatory infiltrate was shown to be inversely proportional to the experimental time intervals (Fig. 2A-E).

**Table 1:**
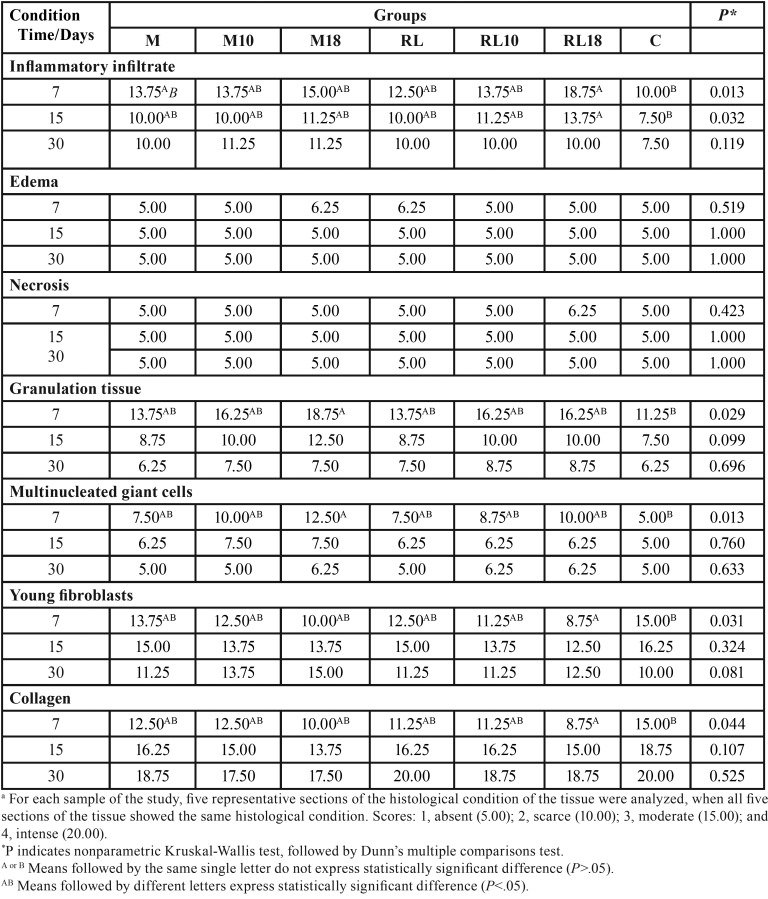
Mean of the scoresa attributed to the cements, after the time intervals of 7, 15 and 30 days, for the seven conditions evaluated.

Circulatory alterations (edema) and tissue degeneration (necrosis) were not expressive and showed no statistical difference among the groups evaluated (*P*>0.05). Granulation tissue was shown to be densely present in Group M18 with significant differences in comparison with the Control Group (*P*=0.029), in the time interval of 7 days (Fig. 2B,E). Multinucleated giant cells were also shown to be more present in Group M18, with statistical difference from Control Group in the time interval of 7 days (*P*=0.013) ( Table 1) (Fig. 2B).

**Figure 3:**
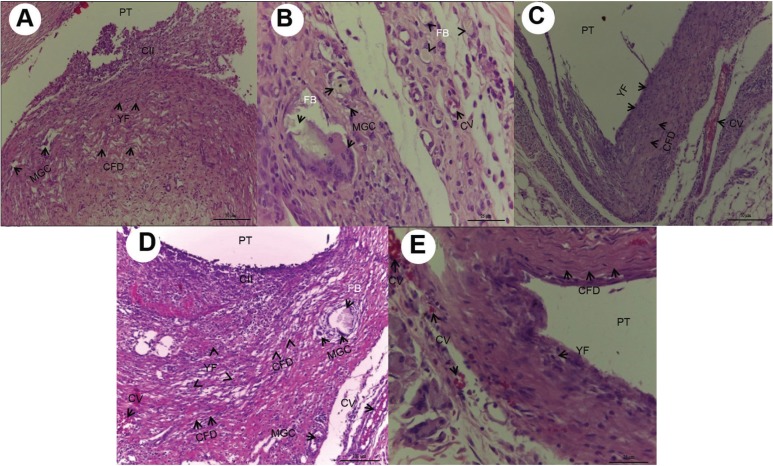
A) 15 days after implantation, Group M10: Chronic inflammatory infiltrate (IIC), deposition of collagen fibers (CFD) disposed in parallel bundles, proliferation of young fibroblasts (YF), and multinucleated giant cells (MGC) (HE, 200Xmagnification, scale: 50µm). Area of polyethylene tube implant (PT). B) 15 days after implantation, Group M18: Multinucleated giant cells involving or close to the foreign body (FB) congested blood vessels (CV) (HE ,400Xmagnifications, scale: 25µm). Area of polyethylene tube implant(PT). C) 15 days after implantation, Group RL10:Deposition of collagen fibers (CFD) disposed in parallel bundles and sometimes dispersed,young fibroblasts (YF),congested blood vessels (CV) (HE, 200X magnification, scale: 50µm). Area of polyethylene tube implant (PT). D) 15 days after implantation, Group RL18: Cavity surrounded by chronic inflammatory infiltrate (CII), congested blood vessels (CV), Deposition of collagen fibers (CFD) and proliferation of young fibroblasts (YF), presence of multinucleated giant cells (MGC) and foreign body (FB) (HE, 100X magnification, scale: 100µm). Area of polyethylene tube implant (PT). E) 15 days after implantation, Group Control: Thick layer of collagen fiber deposition (CFD) disposed in parallel bundles in the midst of scarce young fibroblasts (YF) congested blood vessels (CV) (HE, 400X magnification, scale: 25µm). Area of polyethylene tube implant (PT).

**Figure 4:**
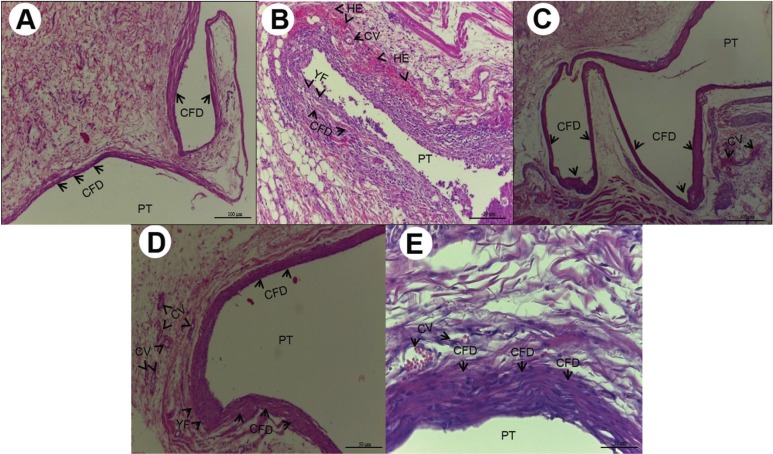
A) 30 days after implantation, Group M10: Thin layer of collagen fiber deposition (CFD) disposed in parallel bundles and dispersed young fibroblasts (HE, 100X magnification, scale: 100µm). Area of polyethylene tube implant (PT). B) 30 days after implantation, Group M18: Deposition of collagen fibers (CFD) sometimes parallel, sometimes dispersed, proliferation of young fibroblasts (YF), Hemorrhagic exudate (HE),congested blood vessels (CV) in the midst of a light chronic inflammatory infiltrate (HE, 200X magnification, scale: 50µm). Area of polyethylene tube implant (PT). C) 30 days after implantation, Group RL10: Cavity surrounded by a thick layer of collagen fiber deposition (CFD), congested blood vessels (CV) (HE, 100X magnification, scale: 100µm). Area of polyethylene tube implant (PT). D) 30 days after implantation, Group RL18: Cavity surrounded by a collagen fiber deposition (CFD), proliferation of young fibroblasts (YF), congested blood vessels (CV) (HE, 200X magnification, scale: 50µm). Area of polyethylene tube implant (PT). E) 30 days after implantation, Group Control: Deposition of numerous layers of collagen fibers (CFD) disposed in parallel bundles involving the cavity, congested blood vessels (CV) (HE, 400X magnification, scale: 25µm). Area of polyethylene tube implant (PT).

In the tissue repair events, Group RL18 demonstrated a smaller quantity of young fibroblasts (*P*=0.031) and collagen fibers (*P*=0.044) in the time interval of 7 days ( Table 1) (Fig. 2D). The quantity of young fibroblasts and collagen fibers increased throughout the experimental time intervals of 15 days (Fig. 3A-E) and 30 days (Fig. 4A-E), without significant difference (*P*>0.05).

-Antibacterial effect

For the same evaluation time interval the inhibition zone measurements were shown to be concentration-dependent; there was significant difference among all the groups relative to the difference concentrations of CHX, for both the Meron and Riva cements (*P*=.001) ( Table 2).

**Table 2:**
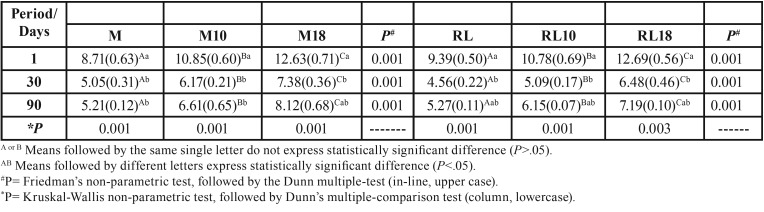
Mean and standard deviation (SD), influence of time and concentration of chlorhexidine digluconate on measures of zones of inhibition.

All the groups demonstrated a reduction in the antibacterial effects from 24h to 30 days, with slight increase in the inhibition zones from 30 to 90 days; there was significant difference between the time interval of 24h and those of 30 and 90 days for Groups M (*P*=0.001) and M10 (*P*=0.001). But there was significant difference between the time intervals of 24h and 30 days for the Riva Cement Groups (*P*=0.001), and for Meron cement, only in Group M18 ( Table 2).

-SBST and ARI 

The SBST showed no statistically significant difference between the Meron Groups of cement (*P*=0.385) after the addition of CHX. For Riva cement, there was significant difference between Group RL-control and Groups RL10 (10%-CHX) and RL18(18%-CHX) (*P*=0.001), demonstrating an increase in the SBST of the cements with the addition of CHX. In the comparison among the different brands of GIC for the same concentration of solution used, there was significant difference only between Groups M-Control and RL-Control (*P*=0.032) ( Table 3).

**Table 3:**
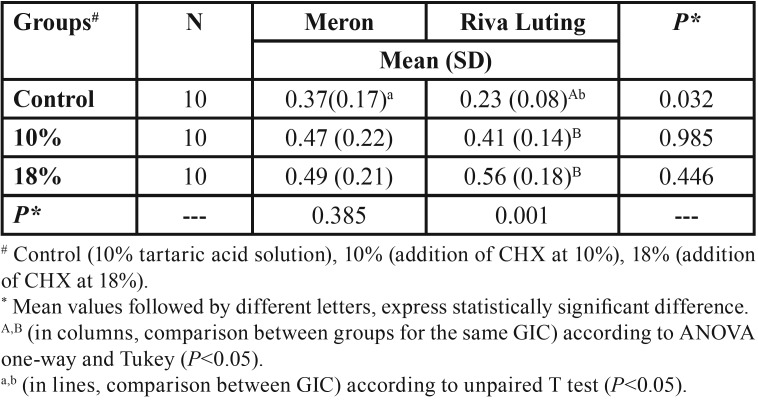
Mean and standard deviation (SD) of the shear bond strength test values of the different groups, expressed in MPa.

The ARI demonstrated that over half of the remnant cement or all of the remnant cement remained on the tooth surface after removing the specimen. The ARI showed no statistically significant difference between the Groups of Meron cement (*P*=0.979) and between the Groups of Riva cement (*P*=0.092), after the addition of CHX.

## Discussion

 In the antimicrobial analysis, a significant increase was verified in the measurements of the inhibition haloes of the cements that contained CHX, suggesting an increase in the antimicrobial effect through the gradual release of CHX. These finding corroborated those of other studies (3,10), and those that have demonstrated (6,10) that a concentration of 10%-CHX was sufficient to provide protection against *S. mutans*, but the increase in this concentration increased the antibacterial effect (6), up to the maximum concentration allowed of 18% (10).

Inhibition haloes were significantly larger in the cements containing the concentration of CHX-18% (10) in studies that verified the addition of CHX for 65 days. The antimicrobial effect remained present during the experimental time interval of 90 days, which demonstrated that the release of CHX also occurred in the long term, and could be important during the entire course of orthodontic treatment.

The SBST probably is the mechanical test that best represents the orthodontic clinical situation (10). The addition of CHX demonstrated no significant difference in SBST for Meron (*P*=0.385), and Riva cement with the addition presented a significant increase (*P*=0.001). These results are in agreement with those of other studies (10,17) that demonstrated that the inclusion of CHX in GICs did not harm the clinical performance relative to possible disturbances such as fracture and/or solubilization, leakages and consequent development of caries and periodontal disease close to the bands. The IRA demonstrated that over half or the entire remnant adhesive remained on the tooth surface after removal of the specimen, irrespective of the addition of the CHX, proving that CHX did not interfere in the bond of GIC to the dental structure.

The focus of the histological analysis was to show the action of GICs on tissues by means of a quali-quantitative evaluation based on aggression to the vascularized live tissue (2). Initial intense inflammatory infiltrate was demonstrated by the two cements, particularly in the Groups with CHX-18%, however, with significant difference only between Riva and the control at 7 (*P*=0.013) and 15 (*P*=0.032) days. Nevertheless, the intensity of the inflammatory infiltrate diminished gradually during the experiment, and in the time interval of 30 days it no longer demonstrated significant difference among the groups. These findings are in agreement with studies (18,19) that showed that CHX is cytotoxic to cells and tissues, and is capable of inducing a significant inflammatory reaction (6,19) even in lower concentrations than those used in this experiment, particularly in the short term.

Human fibroblasts exposed to 0.12% CHX for 30s (20), 1, 5 and 15min (21) and incubated for a period of recovery of 1 day (20) and 7 days (21) respectively, showed no significant cellular recovery, which reduced proliferation by over 70% (21). Although the cytotoxicity of CHX occurs by inhibition of cell protein synthesis (20) and mitochondrial respiration (19), induction of apoptosis at low concentrations, and necrosis at elevated concentrations (19), this cytotoxic potential is related to the time of cell exposure and concentration of CHX (8), but in the GICs this potential may be retarded by the slow release of CHX built into the cement network.

Meron cement with CHX-18% demonstrated a dense granulation tissue and presence of multinucleated giant cells, with significant difference in comparison with the control (*P*=0.029) at 7 days, which corresponded to the body’s response in trying to isolate the foreign body (8,19), however, this condition did not persist in the other subsequent time intervals. This condition was less expressive than that found by other authors (5), who used CHX in a Chip in the subcutaneous tissue of rats, and experiments (5,21) that showed evidence of death of fibroblasts in culture after contact with CHX. This suggests that GIC provided a slow and lower rate of CHX release than the total concentration used in the modification of the cement formula.

The tissue exposed to Riva with CHX in higher concentrations presented a lower tissue repair capacity in the initial time intervals, since Riva-CHX18% demonstrated a lower quantity of fibroblasts (*P*=0.031) and collagen (*P*=0.044) at 7 days, which is in alignment with studies that showed evidence of a reduction in the healing process (2,19) with a reduction in the production of non-collagenous protein and collagen fibers (2). However, the quantities of young fibroblasts and collagen fibers increased during the course of the experimental time intervals (*P*>0.05).

The release of CHX on the GIC surface may have a reinforced antibacterial effect over the course of time, resulting from superficial erosion, exposing a new surface for releasing CHX (6), which allows it to react with cellular structures and lead to direct cell damage or inhibition of bacterial cellular metabolism, due to its substantivity (4,19). On the whole, the addition of CHX to the cements demonstrated to be a highly promising method for obtaining of an antibacterial GIC for orthodontic cementation.

## Conclusions

The antimicrobial effect was demonstrated to be concentration-dependent. The addition of CHX did not negatively influence the SBST and ARI. Meron-CHX-10% was the most biocompatible, and Riva-CHX-18% had more influence on the inflammatory process and presented slower tissue repair.
